# Comparative assessment of Nanotrap and polyethylene glycol-based virus concentration in wastewater samples

**DOI:** 10.1093/femsmc/xtae007

**Published:** 2024-03-05

**Authors:** Kata Farkas, Jessica L Kevill, Rachel C Williams, Igor Pântea, Nicola Ridding, Kathryn Lambert-Slosarska, Nick Woodhall, Jasmine M S Grimsley, Matthew J Wade, Andrew C Singer, Andrew J Weightman, Gareth Cross, Davey L Jones

**Affiliations:** School of Environmental and Natural Sciences, Bangor University, Bangor, Gwynedd LL57 2UW, United Kingdom; School of Environmental and Natural Sciences, Bangor University, Bangor, Gwynedd LL57 2UW, United Kingdom; School of Environmental and Natural Sciences, Bangor University, Bangor, Gwynedd LL57 2UW, United Kingdom; School of Environmental and Natural Sciences, Bangor University, Bangor, Gwynedd LL57 2UW, United Kingdom; School of Environmental and Natural Sciences, Bangor University, Bangor, Gwynedd LL57 2UW, United Kingdom; School of Environmental and Natural Sciences, Bangor University, Bangor, Gwynedd LL57 2UW, United Kingdom; School of Environmental and Natural Sciences, Bangor University, Bangor, Gwynedd LL57 2UW, United Kingdom; UK Health Security Agency, Data Analytics & Surveillance Group, 10 South Colonnade, Canary Wharf, London E14 4PU, United Kingdom; The London Data Company, London EC2N 2AT, United Kingdom; UK Health Security Agency, Data Analytics & Surveillance Group, 10 South Colonnade, Canary Wharf, London E14 4PU, United Kingdom; UK Centre for Ecology & Hydrology, Wallingford OX10 8BB, United Kingdom; Cardiff School of Biosciences, Cardiff University, Museum Avenue, Cardiff CF10 3AX, United Kingdom; Science Evidence Advice Division, Health and Social Services Group, Welsh Government, Cathays Park, Cardiff CF10 3NQ, United Kingdom; School of Environmental and Natural Sciences, Bangor University, Bangor, Gwynedd LL57 2UW, United Kingdom; Food Futures Institute, Murdoch University, 90 South Street, Murdoch, WA 6150, Australia

**Keywords:** concentration methods, enteric viruses, public health, respiratory viruses, sewage surveillance

## Abstract

Wastewater-based epidemiology is now widely used in many countries for the routine monitoring of SARS-CoV-2 and other viruses at a community level. However, efficient sample processing technologies are still under investigation. In this study, we compared the performance of the novel Nanotrap® Microbiome Particles (NMP) concentration method to the commonly used polyethylene glycol (PEG) precipitation method for concentrating viruses from wastewater and their subsequent quantification and sequencing. For this, we first spiked wastewater with SARS-CoV-2, influenza and measles viruses and norovirus and found that the NMP method recovered 0.4%–21% of them depending on virus type, providing consistent and reproducible results. Using the NMP and PEG methods, we monitored SARS-CoV-2, influenza A and B viruses, RSV, enteroviruses and norovirus GI and GII and crAssphage in wastewater using quantitative PCR (qPCR)-based methods and next-generation sequencing. Good viral recoveries were observed for highly abundant viruses using both methods; however, PEG precipitation was more successful in the recovery of low-abundance viruses present in wastewater. Furthermore, samples processed with PEG precipitation were more successfully sequenced for SARS-CoV-2 than those processed with the NMP method. Virus recoveries were enhanced by high sample volumes when PEG precipitation was applied. Overall, our results suggest that the NMP concentration method is a rapid and easy virus concentration method for viral targets that are abundant in wastewater, whereas PEG precipitation may be more suited to the recovery and analysis of low-abundance viruses and for next generation sequencing.

## Teaser

Assessment on the feasibility of a novel, magnetic bead-based extraction of viruses from wastewater for quantitative (PCR-based) and qualitative (sequencing-based) analyses.

## Introduction

Wastewater-based epidemiology (WBE) is an important asset used for providing public health insights for the monitoring of infectious diseases at a community level. Many pathogens, including enteric and respiratory viruses, are excreted in the faeces and urine of infected individuals and, hence, can be isolated from municipal wastewater. The viral concentration dynamics in sewage can indicate the relative abundance of cases within a community (Jiang et al. 2022, Reynolds et al. 2022). WBE has been a valuable auxiliary surveillance tool for those pathogens that are associated with asymptomatic cases. For instance, WBE has been used for the community-level surveillance of poliovirus for decades (Pavlov et al. 2005, Rakoto‐Andrianarivelo et al. 2008, Hovi et al. 2012, O'Reilly et al. 2018, Klapsa et al. 2022). Since the start of the COVID-19 pandemic in 2020, many countries have utilised WBE for quantitative tracking, early warning and variant-level monitoring for SARS-CoV-2 (Ai et al. 2021, Carcereny et al. 2021, Kumar et al. 2021, Wang et al. 2022).

In most wastewater surveillance programmes, sewage samples are taken daily or multiple times a week and transferred to a laboratory for analysis. To utilise the WBE approach successfully, viruses typically need to be concentrated in the samples to enable their detection and quantification. This concentration step can be performed using a range of methods including electronegative/electropositive filtration, ultrafiltration, ultracentrifugation or precipitation with polyethylene glycol (PEG), ammonium sulphate or skimmed milk (Farkas et al. 2020a, Ahmed et al. 2020, Rusiñol et al. 2020, Philo et al. 2021, Kevill et al. 2022). It is important that the concentration method successfully recovers the viruses from the samples while eliminating any impurities that may adversely affect downstream processes, such as nucleic acid extraction, viral detection, and quantification (Ahmed et al. 2022a). In most studies, either quantitative or digital PCR (qPCR or dPCR) are used to quantify the viral genomes (Corpuz et al. 2020; Farkas et al. 2020b, 2020c) and, when sequencing is utilised, this often also requires amplification of the target viruses (Karthikeyan et al. 2022). While PCR-based approaches enable the rapid, sensitive and when needed, strain-level detection of the target, they may be affected by residual organic matter that can interfere with the reverse transcriptase and DNA polymerase enzymes (Ahmed et al. 2022a). However, while dPCR is less affected by such inhibitors (Ahmed et al. 2022b, Jahne et al. 2020, Flood et al. 2021), the equipment required is not available in many WBE laboratories. The viral concentration method should therefore aim to reduce the concentration of organic matter to ensure high quality results, regardless of the detection method used.

Recently, the application of magnetic beads to concentrate viruses in wastewater has been suggested, but only a few studies are available on the development and use of this technique (Karthikeyan et al. 2021, Ahmed et al. 2023, Andersen et al. 2023, Daza-Torres et al. 2023, Feng et al. 2023). The method is quickly and easily performed without the requirement for complex lab equipment and, hence, may be applied for on-site analysis that facilitates the delivery of rapid insights. It is therefore important to improve our understanding of the overall performance of such methods.

In this study, we explored the usefulness of the Nanotrap® Microbiome Particles (NMP, formerly called Nanotrap® Magnetic Virus Particles), for the recovery of different human pathogenic viruses and a faecal indicator virus from wastewater. First, we used samples spiked with the target viruses to estimate recovery efficiency. Then, we performed an intra-laboratory trial using magnetic bead concentration along with PEG precipitation to explore reproducibility. Lastly, we trialled the effect of the magnetic bead concentration method along with PEG precipitation on 42 wastewater samples to investigate sensitivity.

## Methods and materials

### Virus spiking

In order to test the feasibility of the NMP method for virus recovery, ion-exchanged water and wastewater samples were spiked with known concentrations of heat-inactivated SARS-CoV-2 (kindly provided by Prof Richard Stanton, Cardiff University), influenza A/California/07/2009 (H1N1), B/Lee/40 (kindly provided by Dr Eleanor Gaunt, University of Edinburgh), norovirus GII (NoVGII) in diluted and filtered faecal matter from a patient with confirmed infection (kindly provided by Dr Lydia Drumwright, University of Cambridge), measles virus (MeV) in the form of a vaccine (VWR International, USA) and Phi6 bacteriophage, cultured in-house (Kevill et al. 2022). Samples were processed in triplicate.

### Intra-laboratory assessment

To test the reproducibility of the NMP concentration and the PEG precipitation methods, four experienced lab staff members received the same wastewater sample, in triplicate, for each of the methods (NMP, PEG-150, PEG-37.5), resulting in a total of nine identical samples per each of the four individuals. The unspiked wastewater samples, which were processed as detailed below.

### Wastewater samples

For the spiking and intra-laboratory trial, 20 l and 5 l influent wastewater samples were collected using grab sampling at the Bangor wastewater treatment plant (Bangor, Wales) on the 5^th^ Nov 2021 and on the 18^th^ August 2022, respectively. These wastewater samples contained negligible amounts of the target viruses. The samples were processed in triplicate. Further 42 composite wastewater samples were collected, as part of the Welsh National Wastewater Monitoring programme between 28^th^ and 30^th^ November 2022. These samples were processed without replication. The pH, turbidity, electrical conductivity, ammonium and orthophosphate concentrations of the samples were measured as described previously (Hillary et al. 2021, Farkas et al. 2022).

### PEG precipitation

All samples, except those spiked with human viruses, were concentrated using PEG precipitation, as described previously (Farkas et al. 2021). In brief, 200 ml and 50 ml wastewater samples were centrifuged at 10 000 × *g* at 4°C for 10 min and then 150 ml or 37.5 ml of the resulting supernatant was spiked with known quantities of Phi6 bacteriophage as a process control virus. After pH adjustment to 7–7.5, the solution was mixed with PEG 8000 and NaCl to a final concentration of 10% and 2%, respectively. After a 16 h incubation at 4°C, the samples were centrifuged at 10 000 × *g* at 4°C for 30 min and the resulting pellet was subject to nucleic acid extraction. We refer to the PEG method used on high volume (150 ml supernatant) samples as the PEG-150 method, and we use the term PEG-37.5 when the method was applied on low volume (37.5 ml supernatant) samples.

### Nanotrap® Microbiome Particles (NMP) concentration method

The NMP kit was obtained from Ceres Nanoscience Inc., Manassas, VA, USA. At the time of purchase, the product was named Nanotrap® Magnetic Virus Particles and supplied with Nanotrap® Enhancement Reagent 2 (ER2). The kit was used as per the manufacturer's instructions. The samples were centrifuged and spiked, when applicable, as described above. Then, 400 µl of the ER2 buffer was added to 45 ml sample supernatant and vortexed to mix, followed by the addition of 600 µl Nanotrap beads. Samples were inverted to mix and incubated at room temperature for 10 min, which included an inversion at the 5-minute mark. Tubes containing beads were then placed onto a magnetic rack and once the solution became clear with the beads adhered to the side of the tube, the solution was then removed. The beads were recovered in 1 ml molecular-grade water followed by magnetic separation and the removal of the solution. The recovered beads were then subject to nucleic acid extraction.

### RNA/DNA extraction

Viral nucleic acids were recovered from PEG pellets or from NMP concentrate using the NucliSens extraction system (BioMerieux, France) on a KingFisher automated extraction system (Thermo Fisher, USA) as described preciously (Kevill et al. 2022). In brief, the pellets or beads were resuspended in 850 µl Lysis Buffer, mixed and incubated for at least 10 min followed by the addition of the NucliSens magnetic silica beads for DNA/RNA binding. The beads were then washed with NucliSens Wash Buffer #1 and #2 twice and with Wash Buffer #3 once. The nucleic acids were then eluted from the beads in Wash Buffer #3 at 60°C. The final volume of the eluate was 100 µl.

### Virus quantification

The target RNA viruses were quantified using RT-qPCR on a QuantStudio Flex 6 system (Applied Biosystems, USA) as described previously (Farkas et al. 2022). The SARS-CoV-2 N1 gene fragment and phi6 bacteriophage, andthe influenza A and B viruses (FluA and FluB) were assayed in two duplexed qPCR reactions using validated primers and probes (Gendron et al. 2010, CDC 2020, Shu et al. 2021). *Enterovirus* spp. (EV) and norovirus GI and GII (NoVGI and NoVGII) were quantified using a triplex assay while measles virus (MeV) was quantified with a singleplex assay with validated primers and probes (Gregory et al. 2006, Hummel et al. 2006, ISO/TS 2019). In brief, the reaction mixes for RNA viruses contained TaqMan viral 1-step RT-qPCR master mix (Applied Biosystems, Inc., USA), 1 µg bovine serum albumin (BSA), 10 µM forward, 20 µM reverse primers and 5 µM probe. For the duplex SARS-CoV-2/Phi6 and FluA/FluB assays, 16 nmol MgSO_4_ was also added. The amplification was carried out using the following conditions: reverse transcription at 50°C for 30 min followed by enzyme inactivation at 95°C for 20 s, then 45 amplification cycles of 95°C for 3 s, 60°C for 30 s.

CrAssphage qPCR was set up using the QuantiNova low-ROX probe qPCR mix (Qiagen, Germany), 1 µg bovine serum albumin (BSA), 10 µM forward and reverse primers and 5 µM probe (Stachler et al. 2018, Farkas et al. 2022). The reaction conditions were as follows: DNA denaturation at 95°C for 2 min followed by 40 cycles of 95°C for 15 s and 60°C for 1 min.

All samples were run in duplicate and quantification was carried out using a 10^5^–10^0^ genome copies (gc)/µl dilution series of synthetic RNA oligo standards (SARS-CoV-2 and phi6), commercial genomic standards (FluA/B; Twist Bioscience, USA), RNA extracted from MMR vaccine (MeV) or plasmid DNA (NoVGII and crAssphage). Each plate contained multiple non-template controls to assess cross-contamination.

### SARS-CoV-2 sequencing

A subset of SARS-CoV-2 samples processed by the two methods were sequenced (Table S1) to compare the quality of RNA template for variant detection. Following extraction, RNA was purified using a standardised protocol with magnetic bead clean-up of 1.8X Mag-Bind Total NGS beads (Omega BioTek). A LunaScript RT Supermix Kit (New England Biolabs, UK) was then used to synthesise cDNA before sequencing libraries were prepared using NimaGen's EasySeq RC-PCR SARS-CoV-2 whole genome sequencing kit (Nimagen, The Netherlands). The pooled library was spiked with a control (an adapter ligated library supplied by Illumina Inc., San Diego, CA) and run on an Illumina NextSeq 1000 system using a P1 kit (2×150 bp) following concentration loading guidelines provided by Illumina.

### Data analyses

Initial data analysis and quality control for the qPCR data were performed using the QuantStudio Real-time PCR software v1.7 (Applied Biosystems, USA), following MIQE Guidelines (Bustin et al. 2009), with slope between -3.6 and -3.1, efficiency between 90% and 100%. The LOD and LOQ of target viruses has previously been published (Farkas et al. 2022). Sample concentrations were expressed as gc/µl nucleic acid extract. Virus concentrations were transformed to gc/l as follows:


\begin{eqnarray*}
\frac{\rm concentration\,\,of\,\,the\,\,nucleic\,\,acid\,\,extract \times {\rm extract\,\,volume}}{{\rm volume\,\,of\,\,raw\,\,wastewater\,\,processed}}*1000
\end{eqnarray*}

Recoveries for the viruses spiked in wastewater were calculated as:


\begin{eqnarray*}
\frac{\rm viral\,\,concentration\,\,in\,\,the\,\,spiked\,\,sample}{\rm viral\,\,concentration\,\,in\,\,the\,\,spiking\,\,solution}
*100\%
\end{eqnarray*}

CrAssphage recoveries were calculated as:


\begin{eqnarray*}
\frac{\rm concentration\,\,of\,\,the\,\,concentrated\,\,samples}{\rm concentration\,\,of\,\,the\,\,unconcentrated\,\,samples} \times 100\%
\end{eqnarray*}

The full dataset is displayed in Table S1.

The data from duplicate reactions were combined and the average value was used for statistical analysis. Shapiro-Wilk test confirmed that the data were non-normally distributed (*P* < 0.001). The difference among users and methods performance was assessed using Mann-Whitney U test and Kruskal-Wallis tests. Spearman's rank correlation was used to assess the correlation between viral concentrations, recoveries and wastewater physico-chemical properties. Statistical analyses were performed using SPSS v27 (IBM Inc., USA).

We then estimated relative abundance of SARS-CoV-2 lineages of mixed-lineage virus samples in wastewater. The sequencing data were processed using Freyja v1.2.1 (Karthikeyan et al. 2022), which uses Single Nucleotide Variant (SNV) frequency estimation and a depth-weighted demixing tool. Sequencing data quality control (QC) pass rate was determined using the Nextflow implementation of the ARTIC pipeline (https://github.com/connor-lab/ncov2019-artic-nf); a pass is achieved when >50% of the reference sequence (Genbank accession MN908947.3) bases are detected in >10 reads.

## Results

### Spiking experiment

**Figure 1.**
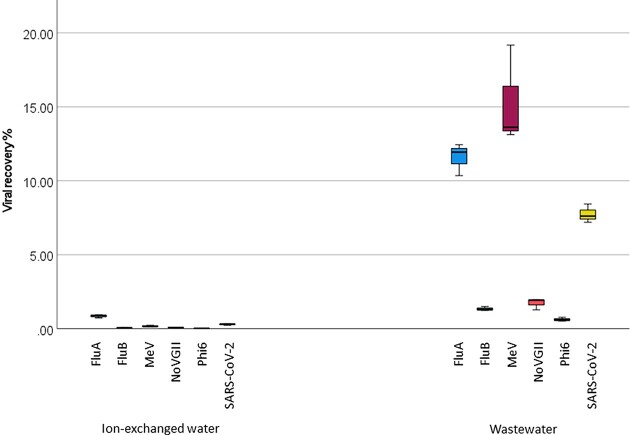
Boxplot comparison of % spiked viral recovery (n=3) for influenza A virus (FluA, blue), influenza B virus (FluB, green), measles virus (MeV, purple), norovirus GII (NoVGII, pink), phi6 bacteriophage (brown) and SARS-CoV-2 (yellow) spiked in ion-exchanged water and wastewater using the Nanotrap® Microbiome Particles (NMP) method. The boxes show the middle 50% of the data set with the horizontal line representing the median value. Error bars represent 95% confidence intervals.

When using the NMP concentration method, significantly higher viral recoveries were obtained from spiked wastewater than spiked ion-exchanged water (Mann Whitney U test; u 33, z-score 6.860, *P* < 0.001, Fig. 1). The pairwise comparison of each virus also gave similar results. The % recovery from wastewater using the NMP method ranged between 0.4 to 21%; the mean recovery was 6% and the median recovery was 4.6%, while for ion-exchanged water the precent recovery range was 0.01 to 1% with a mean recovery of 0.24% and median recovery of 0.12% (Fig. 1). Yields of spiked viruses recovered from wastewater followed the trend: MeV (15.3%) > FluA (11.5%) > SARS-CoV-2 (7.7%) > NoVGII (1.7%) > FluB (1.3%); 10-fold higher on average than yields recovered from ion-exchanged water.

### Intra-laboratory assessment of virus concentration methods

**Figure 2.**
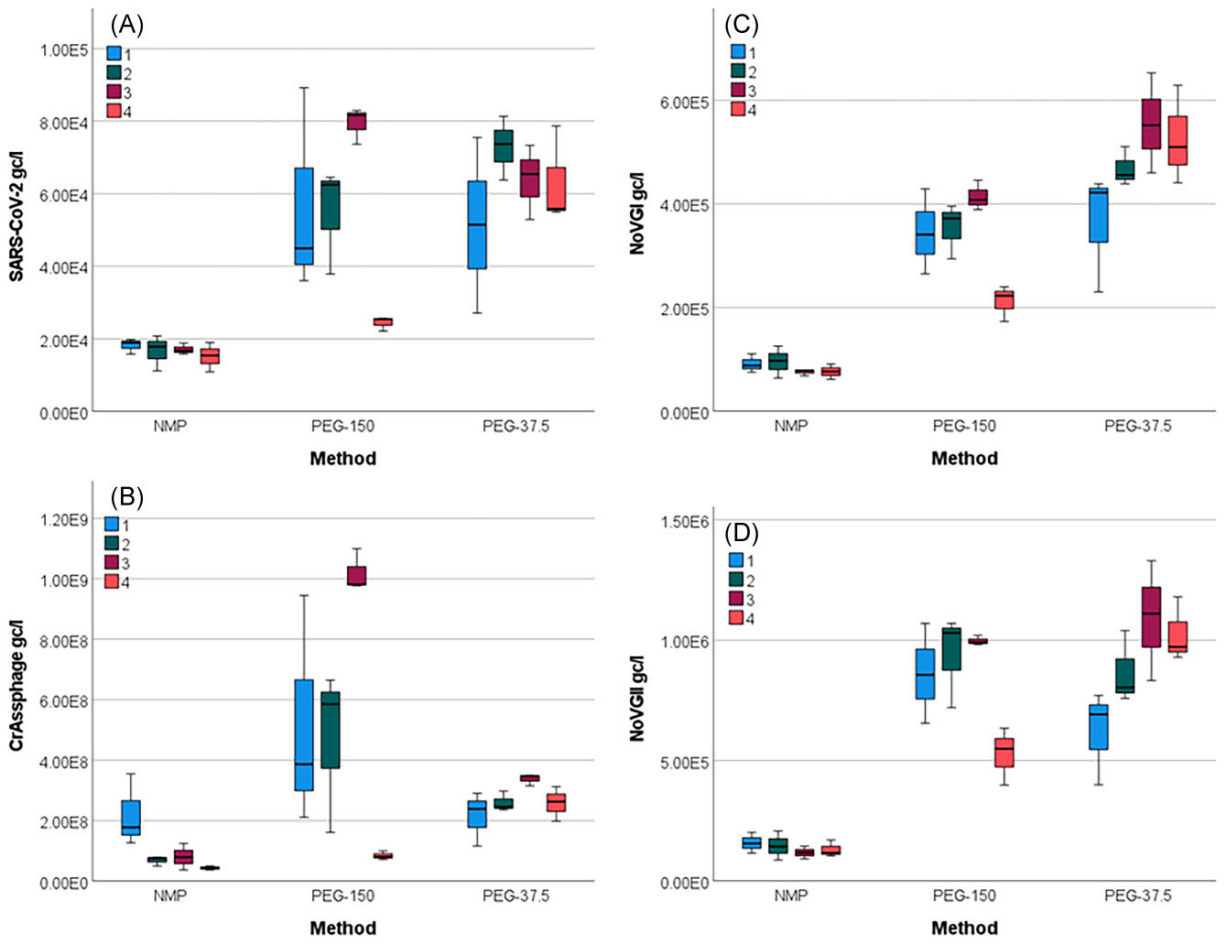
Intra-laboratory trials with four users (1–4) processing 45 ml samples with the NMP method and 150 ml and 37.5 ml samples with the PEG method (PEG-150 and PEG-37.5, respectively) in triplicates for the detection of (A) SARS-CoV-2, (B) crAssphage, (C) Norovirus GI (NoVGI) and (D) Norovirus GII (NoVGII). The boxes show the middle 50% of the data set with the horizontal line representing the median value. Error bars represent 95% confidence intervals.

To assess variability among lab users, four group members processed the same sample in triplicate using the NMP method (45 ml/sample), the PEG-150 (150 ml/sample), and the PEG-37.5 (37.5 ml/sample) precipitation methods. The processed samples were tested for SARS-CoV-2, NoVGI and NoVGII, and crAssphage (FluA/B were tested for but not detected). No significant difference was found in the viral recoveries obtained by the users (Kruskal–Wallis test, *P* >0.05), although User 3 slightly outperformed the others (Fig. 2).

In the same experiment, significant differences were found between the performance of the methods applied for concentration of the different viruses. For SARS-CoV-2, NoVGI and NoVGII, the PEG methods outperformed the NMP method (Kruskal–Wallis test, *P* < 0.001). For crAssphage, the concentrations were significantly lower when the NMP method (*P* < 0.001) and the PEG-37.5 method (*P* = 0.037) were used, compared to the PEG-150 method (Fig. 2B).

### Wastewater testing

For further validation, 42 unspiked raw wastewater samples were processed using the NMP and PEG methods and tested for a range of viruses. FluB virus was not detected in any of the samples with any of the methods, and RSV was only detected in three samples when the PEG-150 method was used (Table 1). The FluA virus detection rates were also substantially higher with the PEG-150 method (62%) compared with those obtained using the NMP method (26%) or the PEG-37.5 method (9%). EV showed the highest detection rates when the PEG-37.5 method was used (26%), whereas the detection rates with the other two methods were lower. SARS-CoV-2 and NoVGI was detected in all samples when the PEG-150 method was applied, and in the majority of the samples using the other methods. NoVGII, crAssphage, and Phi6 were detected in any of the samples regardless of the method used.

**Table 1.** Detection rates (n) for SARS-CoV-2, enteroviruses (EV), norovirus GI and GII (NoVGI, NoVGII), influenza A virus (Flu A), respiratory syncytial virus (RSV), crAssphage, and Phi6 phage process control virus in wastewater samples using the NMP and the PEG methods for concentration.

Method	SARS-CoV-2	EV	NoVGI	NoVGII	FluA	RSV	CrAssphage	Phi6
NMP	98% (41)	14% (42)	98% (42)	100% (42)	26% (42)	0% (42)	100% (42)	100% (41)
PEG—low sample volume	92% (36)	26% (38)	95% (38)	100% (38)	29% (38)	0% (38)	100% (32)	100% (32)
PEG—high sample volume	100% (42)	17% (42)	100% (42)	100% (42)	62% (42)	7% (42)	100% (42)	100% (42)

**Figure 3.**
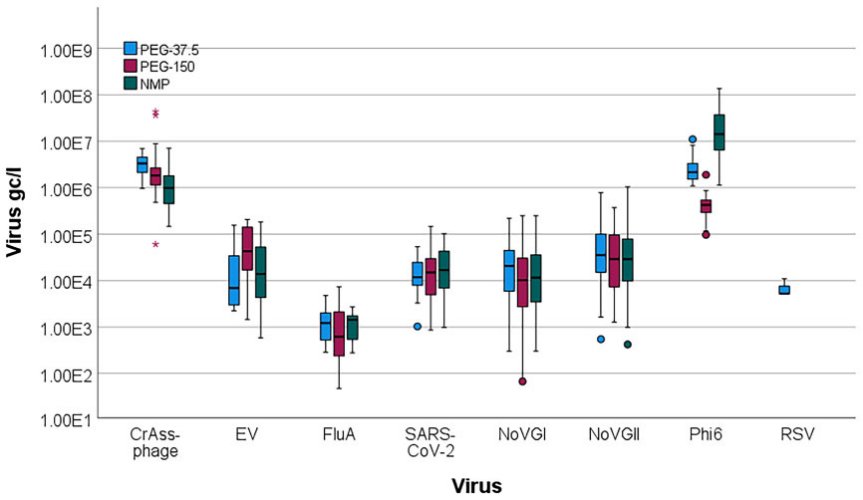
Viral concentrations for crAssphage, enteroviruses (EV), influenza A virus (FluA), SARS-CoV-2, norovirus GI and GII (NoVGI, NoVGII), respiratory syncytial virus (RSV) and Phi6 phage process control virus in wastewater samples using the NMP (green bars) and the PEG-37.5 (blue bars) and PEG-150 (purple bars) methods for viral concentration. The boxes show the middle 50% of the data set with the horizontal line representing the median value. Error bars represent 95% confidence intervals.

Significantly higher virus concentrations were detected for Phi6 when using the NMP method, compared to the PEG-150 and PEG-37.5 methods. No other significant differences were found between methods (Fig. 3).

Overall, the viral concentrations and recoveries obtained by the different methods correlated well for SARS-CoV-2, NoVGI, NoVGII, crAssphage and Phi6 (Table S2). The lack of correlation for EV and FluA may be due to the low detection rates and low virus concentrations. Detection/recovery of SARS-CoV-2, NoVGI, and NoVGII with the different methods also correlated with that of the faecal indicator virus crAssphage. Sample pH showed moderate positive correlation only with NoVGII detection/recovery when low volume of sample was PEG precipitated and a negative correlation was found between pH and crAssphage concentrations, detected when the sample was concentrated using the NMP method. Sample turbidity, electrical conductivity, ammonium and orthophosphate levels showed significant correlations with the human-associated virus concentrations, especially when the PEG-150 method was applied, whereas the viral concentrates derived from the other two approaches mainly correlated with ammonium and orthophosphate levels (Table S2). Interestingly, the Phi6 recovery showed a negative correlation with sample turbidity, electrical conductivity, ammonium and orthophosphate levels.

### SARS-CoV-2 sequencing

Only 14% of samples passed QC for sequencing, comprising 11% processed with the PEG-150 method, and 3% processed using the PEG-37.5 method. All samples processed with NMP magnetic beads failed QC. Low mapping rates of reads to the reference genome meant that the QC threshold was difficult to pass; the percentage of bases that mapped to the SARS-CoV-2 reference genome in samples processed with the NMP beads or the PEG-37.5 method were substantially less than those processed with the PEG-150 method (1.5% vs 3.5%, respectively). Similarly, the average coverage of genome depth was observed for the samples processed using the PEG-37.5 and the NMP methods (144X and 143X, respectively), was substantially less than the 248X average coverage for samples concentrated using the PEG-150 precipitation.

**Figure 4.**
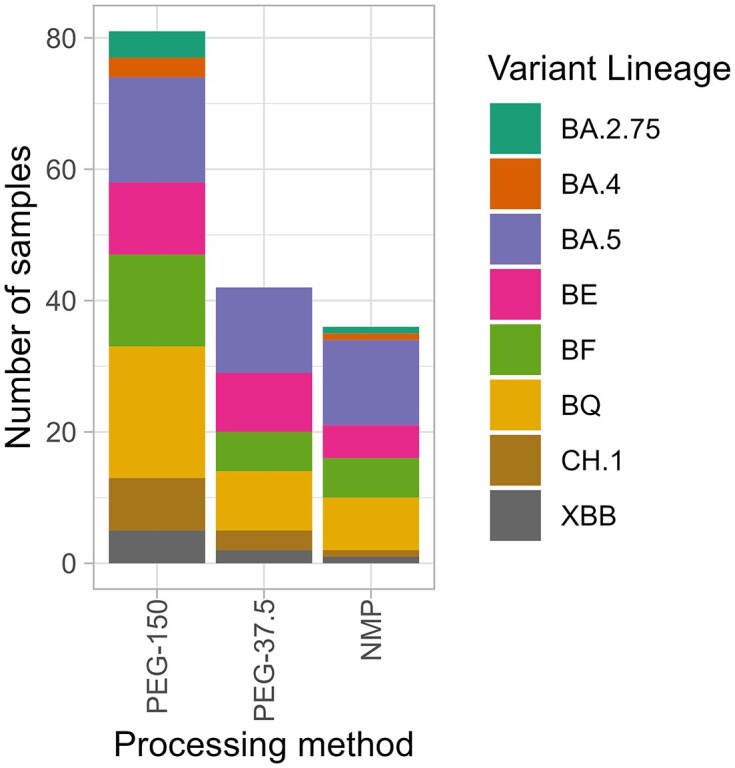
The number of samples at which SARS-CoV-2 lineages were detected using different processing methods. Maximum number of samples calculated as number of samples multiplied by number of variants: 216 samples for PEG-37.5 and NMP, 208 samples for PEG-150. Results include samples that did not pass quality check.

Estimated lineage proportions produced by Freyja were converted to represent the number of samples that successfully detected the lineage when processed with a specific method. Variants were detected across a higher number of sites when samples were processed with either of the PEG precipitation methods (Fig. 4). Samples processed with NMP beads detected variants in the fewest number of samples for all but two variants. One sample processed with PEG failed in library preparations and so was not included in the analyses, therefore variants detected are shown as a proportion of samples successfully processed. General patterns are congruent across methods, such as variant lineage BA.2.75 and BA.5 being the least abundant. However, applying PEG precipitation with high sample volumes detected lineage BQ in 74.1% of cases making it the dominant lineage when quantifying with this method. The other methods detected BA.5 as the dominant lineage.

## Discussion

In this study, we investigated the effectiveness of a novel NMP concentration method versus traditional PEG precipitation for the recovery of viruses from untreated wastewater. Overall, both approaches were suitable for concentrating viruses within wastewater for WBE applications. We found that the NMP concentration method performed better when applied to wastewater than for deionised water (Fig. 1); however, many other virus concentration methods, such as precipitation and ultrafiltration approaches, give higher recoveries in clean water than in wastewater (Farkas et al. 2022). The high NMP recoveries achieved in wastewater may be due to the presence of ions enhancing viral binding to magnetic beads (e.g. viral aggregation, cation bridging). The viral recovery percentiles were comparable with those derived from PEG precipitation and ultrafiltration concentration methods, as determined previously (Farkas et al. 2022) and slightly lower for SARS-CoV-2 compared to a similar study using NMP (Brighton et al. 2024).

We performed an intra-laboratory trial during which well-mixed wastewater subsamples were given to four experienced laboratory technicians to be processed using PEG precipitation methods with high and low sample volumes and using the NMP concentration method. The further steps of sample processing, nucleic acid extraction, and qPCR, were performed in batches by one person using automated systems to reduce human error. Therefore, only the sample concentration step performance was reliant on the different users. No significant differences in users’ performance were noted suggesting that both methods are reproducible (Fig. 2). Some differences amongst users were observed when the PEG-150 method was applied, in which the final viral precipitate is resuspended by elution from the wall of a 250-ml centrifuge bottle. As the viral pellet is often not visible and/or spread on a wide area of the wall of the bottle, it may be hard to completely retrieve; hence, viral recovery may vary. When the PEG-37.5 method is applied, the final pellet is produced in a 50-ml centrifuge tube on a small area of the tube wall, which helps achieve full elution. The NMP method is less reliant on the users’ skills, which is consistent with viral recoveries showing little variation in that element of the intra-laboratory trial. We processed samples in triplicate for each method per user. This generated enough data to assess the useability of the methods, and the limitations of each method has been highlighted. In the comparison of 42 wastewater samples processed with PEG and NMP methods simultaneously the qPCR results suggested that the PEG methods recovered less abundant (i.e. viruses not detected in the majority of wastewater samples in the study) viruses (EV, Flu-A, RSV) more efficiently than the NMP method (Table 1). Furthermore, RSV was only recovered using the PEG method with high initial sample volumes. The viral concentrations for SARS-CoV-2, crAssphage and noroviruses obtained by the different methods correlated well, suggesting that both NMP and PEG methods performed similarly for highly abundant viruses. Interestingly, only the process control virus Phi6 was recovered at significantly higher concentrations with the NMP method compared to the PEG methods, while the quantification of other viruses was not method-dependent (Fig. 3). The Phi6 virus used in this study was derived from an *in vitro* cultured stock (Kevill et al. 2022), which may behave differently from viruses that are abundant in wastewater, as viruses present in wastewater may also bind to the suspended solids.

In most cases, viral concentrations correlated with turbidity and chemical water parameters, suggesting that the more concentrated the wastewater is, the more human viruses can be recovered. However, previous research found that high turbidity has a negative effect on the efficiency of NMP concentration for pepper mild mottle virus (Ahmed et al. 2023), further suggesting that different viruses were recovered at different yields using this method.

We noted similar patterns in the sequencing data for SARS-CoV-2, with PEG-150 being able to detect less abundant variants a greater number of times. The PEG-37.5 precipitation and NMP concentration methods failed to detect several variants in a large proportion of sites, which was correlated to the low average coverage and the low percentage of reads that were mapped. It is unusual to have low coverage across all methods; all samples were sequenced together on one run to allow comparisons to be made between processing methods, regardless of run chemistry. However, separating runs based on methods would not only have given better coverage (increasing the chance of samples passing the QC threshold) but would also have removed the possibility that a processing method may carry inhibitors (e.g. chemicals or organic matter) that affect the overall run. While all methods were able to distinguish between the different SARS-CoV-2 variants in samples, PEG-150 precipitation demonstrated the greatest ability to do this repeatedly and at low virus abundance, While more optimisation is still needed to improve the success rate, the increased sensitivity for detecting variants demonstrates the potential of this approach.

## Conclusions

Overall, our results show that the NMP method is suitable for certain WBE applications. For example, in situations where the speed of results (rapid need for determination of presence or absence, for instance) outweighs the need for detailed quantification, the beads offer a rapid concentration method, which can be automated if the sample volume is lowered to 10 ml, enabling high throughput testing (Karthikeyan et al. 2021, Brighton et al. 2024), however, small sample volumes may prevent the detection of low abundant viruses. For cases where quantification is important, our results show that PEG precipitation applied on high-volume samples is better able to detect less abundant viruses in RT-qPCR, and facilitates the detection of a greater range of variants in SARS-CoV-2 variant sequencing.

## CrediT authorship contribution statement

Kata Farkas (Conceptualization, Methodology, Writing & editing, Funding acquisition, Supervision), Jessica Kevill (Conceptualization, Methodology, Investigation, Formal analysis, Writing & editing, Supervision), Rachel Williams (Methodology, Formal analysis, Writing & editing, Supervision), Igor Pântea (Methodology, Investigation, Writing & editing), Nicola Ridding (Methodology, Investigation, Writing & editing), Kathryn Lambert-Slosarska (Methodology, Investigation, Writing & editing), Nick Woodhall (Methodology, Investigation, Writing & editing), Jasmine M.S. Grimsley (Conceptualization, Writing & editing, Funding acquisition), Matthew J. Wade (Conceptualization, Writing & editing, Funding acquisition), Andrew Singer (Writing & editing, Funding acquisition), Andrew J Weightman (Writing & editing, Funding acquisition), Davey L. Jones (Conceptualization, Methodology, Writing & editing, Funding acquisition, Supervision).

## Supplementary Material

xtae007_Supplemental_File

## Data Availability

Metadata is available in Supplementary Table S1.
